# Quantitative Determination of Trace Heavy Metals and Selected Rock-Forming Elements in Porous Carbon Materials by the X-ray Fluorescence Method

**DOI:** 10.1021/acsomega.1c03217

**Published:** 2021-09-15

**Authors:** Vladimir G. Povarov, Tatyana N. Kopylova, Maria A. Sinyakova, Viacheslav A. Rudko

**Affiliations:** † Saint Petersburg Mining University, 2, 21st Line, Saint Petersburg 199106, Russia; ‡ Saint Petersburg State University, 13B Universitetskaya Emb., Saint Petersburg 199034, Russia

## Abstract

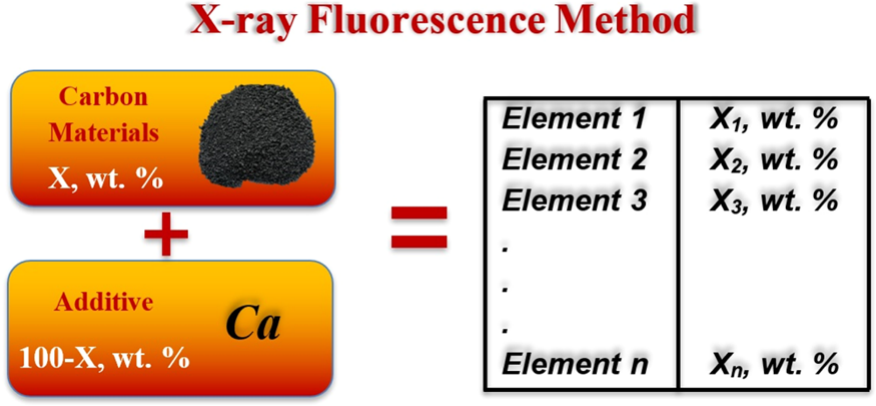

A new X-ray fluorescence (XRF) method is proposed for sample preparation and impurity quantification for elements heavier than sodium in carbon materials. The analysis is suitable for various materials including amorphous ones, such as petroleum cokes, with an impurity content higher than 0.01%. We compared a new method with the regular additive method to measure impurities in electrode graphite and petroleum coke. The XRF-based method provides the same sensitivity and accuracy and much greater reproducibility of the analysis results for variations in the sample mass, its density, and coverage by exciting X-ray radiation. The method does not require changes in the instrument software and is easily implemented on commercial analytical equipment.

## 1 Introduction

It is often necessary to quantitatively determine the impurities of heavy metals and rock-forming elements in materials of various nature, without directly destructing them.^1−5^ A certain approach is required when working with carbon-based materials.^6−8^ Most often, in this case, one has to deal with soot,^9^ graphite,^10−12^ nanotubes, petroleum cokes, and coals.^13−15^ Typical values of the content of trace elements in carbon materials of various types are in limits 10–1500 ppm.^16^

The classic approach to solve this problem is to preburn the sample, then dissolve the residue in a mixture of concentrated acids, and determine the elements using one of the conventional analysis.^17−19^ This method has two disadvantages. First, sample preparation requires a specially equipped workplace.^20−22^ Second, many elements (mercury, lead, or sulfur) partially volatilize during combustion.^23−25^ These problems limit the use of the methods for educational purposes in educational and scientific laboratories.^26^ The only method for the rapid and direct detection of elements heavier than sodium without prior destruction of the material is X-ray fluorescence (XRF). The corresponding devices are widely used in research and industrial laboratories.^27,28^ However, their use is complicated by the low sensitivity of modern detectors to the radiation of carbon atoms, which does not allow one to reliably determine its content from the intensity of the characteristic radiation.^29,30^ It is necessary to plot calibration dependencies for each element to be determined,^28,31,32^ which greatly increases the time and cost of the analysis. The variant of solving this problem proposed by the authors is based on the use of the method of additives but differs in the method of calculation. It is highly resistant to variations in sample preparation and allows us to find the absolute content of all detected elements by adding one of them.

This article is devoted to the practical application of the said method and its comparison with the classical variants.

## 2 Theoretical Section

Let us assume that we have a common XRF analysis data of a certain carbon material for the content of elements heavier than sodium in the fundamental parameter mode (FPM1). Samples should be homogeneous only. Since carbon is not determined in such an analysis, the content of other elements is normalized either to 100% or another predetermined value. It means that we can calculate only the ratio of elements in a sample but not the absolute content. Then, we add to the initial sample a known amount of an element (e.g., in the form Fe_2_O_3_, KCl, CaCO_3_, etc.) and again perform XRF (FPM2). Now with the known mass of the sample and the added element, we can calculate the absolute content of all the elements in the sample.

Indeed, let the mass of the sample of material be *M*_0_ and the mass of the added element A be denoted as *m*_a_ (*m*_a_/*M*_0_ < 0.01). The mass fractions of elements A, B, C, and so forth in the FPM1 are denoted as *X*_A_, *X*_B_, *X*_C_, and so forth, and *Y*_A_, *Y*_B_, *Y*_C_, and so forth in the FPM2 results. We denote the true mass fractions of elements in the material as *Z*_A_, *Z*_B_, *Z*_C_, respectively. If the added element was not contained in the original sample (*X*_A_ = *Z*_A_ = 0), then for any other element B (present in the initial material), the following can be written
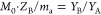


It means that the ratio of the masses of the elements B and A in the sample with the additive is equal to the ratio of their mass fractions according to FPM2 analysis results. As a result, we obtain
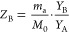
1Here, the XRF of the material without additive is only needed to select the element A that is absent in the sample.

If it is impossible or undesirable to select an additive element that is absent in the original sample, then the calculation becomes more complicated. In this case, we can use one of the two approaches. The first is the traditional application of the additive method. Indeed, if the values of the analytical signals of the element A are known in both samples (without the additive *I*_1_ and with the additive *I*_2_), then the true content of element A in the original sample is found by the classical formula^28^
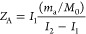
2

The mass fractions of the remaining elements can be calculated from the proportions of the form
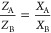
3

A serious disadvantage of this method is the high sensitivity of the analysis results to sample preparation, the location of the sample under X-ray beam, and the heterogeneity of the surface density of the sample.

The second method is free from this disadvantage. When calculating the composition, two elements are used: an additive A and another element B, which is also initially present in the sample. For a sample with additive A, we can write down .

On the other hand, the value of the ratio *Z*_B_/*Z*_A_ = *X*_B_/*X*_A_ = *g* is known to us from the first analysis FPM1 (with *g* > *f* due to the addition of the element A).

Then, we obtain for *Z*_A_
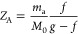
4

The advantage of the latter formula over 2 is the use of mass fractions, rather than absolute values of intensities. These ratios depend neither on the surface density of the analytes nor on the degree of sample dilution with fluxes or the uniformity of the sample density. To calculate the true contents of other elements, as in the first method, formula 3 is used.

## 3 Experimental Section

All measurements were performed on a Shimadzu XRF-1800 XRF wave spectrometer. The cathode current was 90 mA; the tube voltage was 40 kV. The aperture was 20 mm. The calculations were performed using the method of fundamental parameters (qualitative–quantitative method) using a standard algorithm for taking into account the effect of the sample matrix (carbon) on the absorption of X-ray radiation. The values of 0.1% recommended by the manual of device for elements in the Na–K series and 0.01% for Ca–U elements were taken as the level of detection (LOD). In this case, the ratio signal/noise should not be lower than 10. Usually, when analyzing carbon samples, the real LOD of some elements turns out to be lower than the recommended one since the contribution of carbon to the noise of the characteristic radiation is much less than the contribution of silicon, aluminum, and other typical elements of rocks.

**Figure 1:**
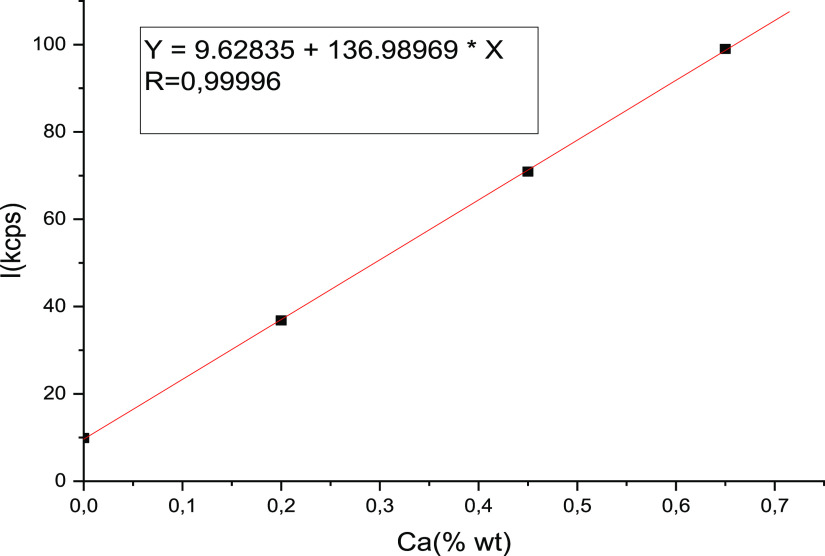
Intensity plot of X-ray calcium (Kα line) vs percentage calcium in graphite.

Formulae 1–4 are true when the intensity plot versus the content of additives is linear. In Figure 1, we can see linear dependence.

A sample of electrode graphite was used as a model object. A 50 g sample was ground in a ZrO_2_-coated ball mill and then thoroughly mixed. When a sample of graphite was calcined in air at 900 °C for 3 h; it was found that the mass of the residue was 0.61%. For this reason, to take into account the matrix effect of absorption of fluorescent radiation, the carbon content in graphite was taken equal to 99%. For each measurement, we took a separate sample of graphite weighing ≈0.1 g. The analysis results of three samples are presented in Table 1.

**Table 1:** Content (% wt) of Impurities in Three Weighed Portions of the Initial Graphite According to the Data of XRF Analysis^a^

element	*X*_1_	*X*_2_	*X*_3_	*X̅*	Δ_1_^b^
Fe	0.652	0.647	0.650	0.650	0.003
Ca	0.196/9.9	0.183/10.4	0.184/9.7	0.188/10.0	0.007/0.3
S	0.075	0.083	0.085	0.081	0.005
Al	0.041	0.048	0.043	0.044	0.004
Si	0.035	0.039	0.038	0.037	0.002
Ca/Fe	0.300	0.283	0.283	0.289	0.010

a The carbon content is taken equal to 99% wt. Herein after, data for Ca are presented (% wt/fluorescence intensity).

b is the standard deviation of a single measurement.

It should be kept in mind that the residue after calcination is represented by oxides, sulfates, and silicates, while in the initial graphite, the same elements can be in the form of carbides, silicides, or sulfides. Therefore, the loss of mass during combustion can be used to estimate the carbon content when taking into account the absorption of fluorescent radiation from the detected elements but not to accurately determine the content of these elements.

We used calcium as an additive. It was taken in two forms: CaCO_3_, reagent grade, and a solution of CaCl_2_ in isopropanol (4.5 mg of Ca/mL) with the addition of glycerin 1% wt. A weighed portion of carbonate was thoroughly ground in an agate mortar along with a weighed portion of the initial graphite. Then, 100–200 mg of the mixture was evenly placed on the surface of the steel substrate within a circle with a diameter of 3 cm, 4–5 g of cellulose was poured on top of the sample, and a tablet for analysis with a diameter of 30 mm was prepared by pressing on a hand press with a force of 25 tons. This initial sample analysis showed that the value of the analytical signal from the CaCO_3_ additive was reproduced with a relative error of at least 20%. We assumed that the mixing of the two solid phases is not completely homogeneous since the amount of the additive phase is much lower than the amount of the graphite. It is obvious that when the calcium content in the initial graphite is 2–10 mg/g, the weight of the sample of calcium carbonate should be 5–25 mg per 1 g of graphite. Mixing such different amounts cannot be guaranteed to be uniform. On the other hand, graphite has a highly porous structure, and if its sample is impregnated with an amount of a calcium salt solution of a similar mass, with the addition of a viscous nonvolatile liquid (glycerin), then after processing the emulsion in an ultrasonic bath and evaporating the solvent, the graphite particles will be uniformly impregnated with the calcium salt. In other words, it was decided to use the classical technique of applying liquid chromatographic phases for packed columns.^33^Table 2 shows the analysis results for five graphite samples of 1 g with additions of 4.5 mg Ca in the form of CaCl_2_.

**Table 2:** Content (% wt) of Ca and Fe in Graphite after the Addition of 4.5 mg Ca per 1 g of Initial Graphite According to XRF Analysis^a^

no.	Ca	Fe	Ca/Fe
1	0.629/70.90	0.278	2.26
2	0.614/72.5	0.295	2.08
3	0.603/65.3	0.302	2.00
4	0.611/69.5	0.288	2.12
5	0.605/70.6	0.305	1.98
±Δ_1_	0.61 ± 0.01/69.8 ± 2.7	0.294 ± 0.01	2.09 ± 0.11

a The carbon content is taken equal to 99% wt.

Table 3 shows the results of the analysis of the same five samples from Table 2 but under conditions when the graphite “spot” on the tablet was forcibly displaced in an arbitrary direction relative to the hole in the steel cover of the sample holder. In this way, we simulated uneven mixing and uneven layer thickness.

**Table 3:** Content (% wt) of Ca and Fe in Five Graphite Samples after the Addition of 4.5 mg of Ca per 1 g of Initial Graphite under Conditions of Random Displacement of the Sample Relative to the Irradiation Area^a^

no.	Ca	Fe	Ca/Fe
1	0.612/64.8	0.280	2.19
2	0.609/51.9	0.295	2.06
3	0.550/33.2	0.300	1.83
4	0.573/59.7	0.284	2.02
5	0.570/58.2	0.280	2.04
*X* ± Δ_1_	0.58 ± 0.03/54 ± 12	0.288 ± 0.01	2.03 ± 0.13

a The notation and normalization conditions are the same as in Table 2.

The relative standard deviation of a single measurement of most of the values presented in Tables 1–3 is in the range of 0.4–10%. However, for the calcium fluorescence intensity from Table 3, it is about 24%. This intensity was used in calculations in the classical formula of the additive method (formulae 2).

It is clear that the greater error of the fluorescence intensity is a direct consequence of the forced displacement of the graphite samples relative to the region of registration of the characteristic XRF on the substrate surface.

## 4 Results and Discussion

**Figure 2:**
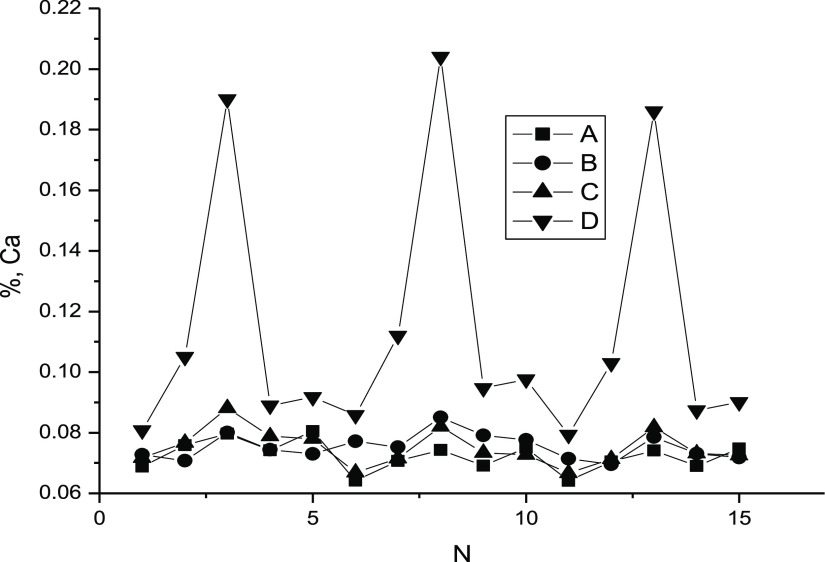
Calcium content in graphite according to Tables 1–3: A—calculation according to the formula 4, Tables 1 and 2; B—calculation by formula 2, Tables 1 and 2; C—calculation by formula 4, Tables 1 and 3, D—calculation by formula 2, Tables 1 and 3.

The results of calculating the calcium content in graphite according to Tables 1–3 are presented in Figure 2. It should be kept in mind that in sum, we had 3 × 5 = 15 combinations of the form (composition from Table 1—composition from Table 2 or 3). The combinations are equivalent, and they were always analyzed in the ascending order of the numbers of the compositions in both tables. In this case, the compositions from Tables 2 and 3 were sorted out with a fixed composition from Table 1.

Comparing the results of calculations using different formulas with different combinations of initial data, it can be seen that both the classical method of additives (formula 2) and the method of relative concentrations (formula 4), when using the data in Tables 1 and 2, give almost identical results. A slightly higher error when calculating using formula 4 is expected since the calculation uses the concentrations of two elements and not one. The fundamental difference between the methods appears when using the data from Table 3. In this case, the classical formula of the additive method leads to unreasonably large errors. This is especially noticeable when using the data for the third composition from Table 3. The latter is not surprising since the calcium fluorescence intensity for this measurement is 2 times lower than that presented in Table 2. Nevertheless, the calculation with the same data according to formula 4 does not lead to discrepancies with the calculations by both formulas when using the data from Tables 1 and 2. Table 4 shows the results of averaging the obtained values of calcium concentration in graphite for all four sets of Figure 2.

**Table 4:** Results of Statistical Processing of Measurements of Ca Content in Graphite by Variants A, B, C, and D

calculation option	average Ca content, %	Δ_1_	*K*^a^, %, *n* = 15, *p* = 0.95
(A) formula 4, Tables 1 and 2	0.072	0.005	3.8
(B) formula 2, Tables 1 and 2	0.075	0.004	2.9
(C) formula 4, Tables 1 and 3	0.075	0.006	4.4
(D) formula 2, Tables 1 and 3	0.113	0.043	21

a , *t*(*n*, *p*) is Student’s coefficient, *t*(15, 0.95) = 2.131.

Comparison of the results presented in Table 4 was performed using Fisher’s test.^34^ The maximum ratio of the squares of the sample variances of the measurement results for options A–B–C is (4.4/2.9)^2^ = 2.3. With the number of degrees of freedom in both series equal to 14 and a significance level of 0.05, the tabular value of the Fisher test is 2.5. This is, albeit not much, but more than the ratio of sample variances. Therefore, the measurement for options A, B, and C can be considered equivalent. Calculations for the variants D and C (the minimum of all possible for the sets with the participation of the D series) give the ratio of the squared sample variants equal to 22.8. This is much more than 2.5. Therefore, these measurements are not equally accurate.

After averaging the calcium content from Table 4 for variants A, B, and C, we obtain the average value of the calcium content in the graphite sample 0.074%. Then, using formula 3 and the data of Table 1, we find the contents of the remaining elements: Fe—0.26%, Si—0.015%, Al—0.017%, and S—0.032%.

Another important example of the application of the method under consideration can be obtained in the analysis of petroleum carbon materials, namely, petroleum cokes of an amorphous structure. They are obtained as a result of thermal polycondensation reactions of heavy distillate or residual raw materials at a temperature of about 500 °C in an oxygen-free environment. Their main impurity is always sulfur. When petroleum coke is burned, it volatilizes in the form of SO_2_, and therefore, the total impurity content cannot be estimated in this way. Transition elements such as silicon and aluminum are also found. Table 5 shows the results of the primary analysis petroleum coke sample by XRF performed under the conditions described previously. The content was calculated according to formula 4.

**Table 5:** Primary Result of Analysis of a Coke Sample^a^

element	% wt at 99% C	% wt (formula 4) at 99% C	% wt (formula 4) at 95.98% C	% wt (formula 4) at 97.09% C	% wt (formula 4) at 96.87% C
S	0.9591	3.51295	2.77029	2.98773	2.93417
Fe	0.1070	0.39191	0.04168	0.04095	0.04109
Ca	0.0089	0.03260	0.03257	0.03252	0.03256
V	0.0067	0.02454	0.02521	0.02496	0.02501
Si	0.0046	0.01685	0.01261	0.01394	0.01353
total	0.9900	3.97838	2.87771	3.09649	3.04665

a After adding 2 mg of calcium ions to 1 g of a coke sample, the sulfur content was 0.9379%, and the calcium content was 0.0621%.

It is important that in the first calculation using formula 4 (column 3, Table 5), we obtain the total impurity content of 4.015%. Therefore, the carbon content is equal to 96, and not 99%, as we initially assumed. Since the role of carbon is reduced to scattering and absorption of X-ray radiation (both primary, from the source, and secondary, from other elements of the sample), in modern programs for processing the results of XRF, there are algorithms for accounting for this effect. Yet, the values of 96 and 99% seemed too close to be of concern. However, it turned out that if the carbon content in the petroleum coke is left at 99%, then, overestimated sulfur contents are obtained. The point is that insignificant variations in the carbon content in petroleum coke are accompanied by large variations in the content of impurities, especially sulfur.^35^ Also, if this circumstance is ignored, then the ratio of the concentrations of these elements will be found incorrect, and since they are included in formula 4, then the absolute content turns out to be incorrect. In this case, you need to repeat the calculation, with new carbon content. In our case, it will be 96%. Next, we need to find the concentration of all impurities again using formula 4, sum them up, and calculate the new carbon content. As it turned out, this procedure converges quickly. Usually, one recalculation is sufficient (column 4, Table 5). To illustrate the nature of the convergence, Table 6 presents the results of three iterations. The amount of carbon is indicated in the column headings.

**Table 6:** Results of XRF Analysis of Metallurgical Coke Standard Sample USA (Passport Value for S = 0.75 ± 0.05%)

trace elements	% wt initial analysis	% wt initial analysis (coke + Ca)	% wt iteration N3	% wt iteration N3 (coke + Ca)	% wt corrected for oxide form
Si	0.7132	0.6163	1.7858	1.5282	1.77477
Al	0.4088	0.3479	1.1457	0.7936	1.13862
Fe	0.3493	0.2373	0.984	0.8334	0.97792
S	0.2962	0.2351	0.802	0.6265	0.79705
K	0.0722	0.0483	0.2051	0.1422	0.20383
Ca	0.0466	0.0826	0.1355	0.2481	0.13466
Ti	0.0278	0.0178	0.083	0.0553	0.08249
Na	0.0243	0.0222	0.0575	0.0524	0.05714
Mg	0.0238	0.0194	0.0567	0.046	0.05635
Cr	0.0121	0.0089	0.0369	0.0283	0.03667
Cl	0.0107	0.3535	0.0301	0.9821	0.02991
Ba	0.0081	0.0047	0.0242	0.0147	0.02405
Ni	0.0042	0.0033	0.0162	0.0125	0.0161
C	98	98	94.63	94.63	89.63
ash					10.37

As a final check, a standard sample of metallurgical coke supplied by LECO (PN 502-683, LN 12293, Prox-Plus Metallurgical Coke Reference Material) was analyzed to calibrate the C–H–N–S elemental analyzers. An XRF analysis of a coke sample was performed. The first sample was the original coke. A second sample was obtained by adding a known amount of calcium to the original coke. For this, 1 mL of a CaCl_2_ solution in isopropanol was added to a sample of coke mass of 1.05 g. The concentration of calcium ions in the solution was 2 mg/mL. Then, the sample was dried at 140 °C and mixed with a sharpened Teflon stick. The results of analyzes of both samples are presented in Table 6 in columns 2 and 3, respectively. Moreover, in both cases, the total carbon content was randomly taken to be equal to 98% of the mass to see how the iterative algorithm would work. It turned out that three iterations are quite enough to reach an acceptable level of convergence. The corresponding results are presented in columns 4 and 5 of Table 6. The sulfur content was overestimated compared to the passport value.

In addition to sulfur, the sample contains several other impurities. Moreover, these impurities are in the oxide form and not in the form of compounds with carbon. Naturally, when these elements are converted into oxides, the total mass of the sample increases, and the mass fraction of carbon, like other elements, decreases. The composition of the sample after this manipulation is presented in the last column of Table 6. Unfortunately, the sample documentation provides only few values available for measurement by the discussed method—sulfur content (0.75 ± 0.05%), the total carbon content (87.8 ± 0.7%), and ash content (9.89 ± 0.18%). These values calculated by us were 0.8% for sulfur, 89.6% for total carbon, and 10.4% for ash. The slight excess in the found values can be explained by the absence of corrections for the content of nitrogen (1%) and hydrogen (0.14%) in the sample. Naturally, in the calculation, we should not use these sample passport data. Nevertheless, when using the described method, a preliminary determination of the composition of the matrix always makes sense.

A laboratory sample of coke obtained by pyrolysis of fuel oil was a second sample. A portion of coke was ashed. The mass of the ash was 2%. This ash was fused with NaOH at 500 °C and then dissolved in water. The content of iron and silicon in the resulting solution was determined by photocolorimetry. Iron was determined in the form of a complex with *o*-phenanthroline and silicon in the form of reduced silicomolybdic acid (blue form). Then, the results were compared with the data obtained by the above-described XRF method. The results were highly similar in all cases. Therefore, for iron, a content of 0.09% was obtained by XRF and 0.1% by photocolorimetry. For silicon, the values were 0.13 and 0.14%, respectively.

## 5 Conclusions

The proposed version of the XRF determination of impurities in carbon materials technically does not differ from the classical additive method used in XRF analysis. However, the use in calculations of the values of relative concentrations instead of the values of the absolute fluorescence intensities of the additive element makes it possible not only to minimize the mass of the solid sample but also to eliminate the influence of local inhomogeneities in the composition and location of the sample on the analysis results. It is easy to see that in a similar way, it is possible to determine the content of the above elements in the samples of plant or polymer origin, provided that they have pores, which is of great importance for solving problems of environmental monitoring.
